# An ultrahigh-fidelity 3D holographic display using scattering to homogenize the angular spectrum

**DOI:** 10.1126/sciadv.adi9987

**Published:** 2023-10-12

**Authors:** Jiamiao Yang, Lei S. Li, Qiaozhi He, Chengmingyue Li, Yuan Qu, Lihong V. Wang

**Affiliations:** ^1^ School of Electronic Information and Electrical Engineering, Shanghai Jiao Tong University, Shanghai 200240, China.; ^2^ Caltech Optical Imaging Laboratory, Andrew and Peggy Cherng Department of Medical Engineering, Department of Electrical Engineering, California Institute of Technology, Pasadena, CA 91125, USA.; ^3^ Institute of Marine Equipment, Shanghai Jiao Tong University, Shanghai 200240, China.

## Abstract

A three-dimensional (3D) holographic display (3DHD) can preserve all the volumetric information about an object. However, the poor fidelity of 3DHD constrains its applications. Here, we present an ultrahigh-fidelity 3D holographic display that uses scattering for homogenization of angular spectrum. A scattering medium randomizes the incident photons and homogenizes the angular spectrum distribution. The redistributed field is recorded by a photopolymer film with numerous modulation modes and a half-wavelength scale pixel size. We have experimentally improved the contrast of a focal spot to 6 × 10^6^ and tightened its spatial resolution to 0.5 micrometers, respectively ~300 and 4.4 times better than digital approaches. By exploiting the spatial multiplexing ability of the photopolymer and the transmission channel selection capability of the scattering medium, we have realized a dynamic holographic display of 3D spirals consisting of 20 foci across 1 millimeter × 1 millimeter × 26 millimeters with uniform intensity.

## Teaser

Discover an ultrahigh fidelity 3D holographic display through scattering medium.

## INTRODUCTION

A three-dimensional (3D) holographic display (3DHD) can present the volumetric information of an object by recording both the amplitude and phase of the optical field. Because it enables autostereoscopy of a true 3D object from different perspectives, adding binocular perception of 3D depth, 3DHD is regarded as the ultimate implementation of the 3D display (*1*–*4*). Since it was first reported by Gabor (*5*) in 1948, holography has been widely explored for 3D display. Nowadays, static 3DHD is widely used in museum exhibitions, allowing close observation of virtual replicas of precious objects. Dynamic 3DHD can display virtual objects vividly and in motion, which is crucial to virtual reality, augmented reality, and the metaverse, and will promote the development of intelligent manufacturing, remote education, telemedicine, anticounterfeiting, and entertainment (*6*–*9*).

3DHD has two major implementations: one is based on spatial light modulators (SLMs), and the other is based on holographic materials (*10*, *11*). In SLM-based 3DHD, depending on the object to be displayed, the hologram is first calculated based on either a look-up table algorithm, a tilted angular spectrum model, or a deep neural network. Next, the hologram is loaded onto an SLM, such as a liquid crystal SLM, a digital micromirror device (DMD), or an acousto-optic modulator, which modulates a coherent reference light and then generates a virtual image (*12*–*19*). Unfortunately, current SLMs are severely limited in their modulation modes, pixel sizes, and phase continuity; thus, they can generate only a low-quality approximation of the desired optical field, with poor spatial resolution and severe color distortion (*20*). Yu *et al.* (*21*) proposed controlling the volume speckle fields to increase the spatial resolution to a few micrometers. However, this method still suffers from the limitation of SLMs, making it difficult to improve the fidelity further and to modulate an information-rich hologram. Metasurfaces with a compact subwavelength scale provide potential materials for next-generation holographic display (*22*). By manipulating parameters such as the rotation angle of meta-atoms within the metasurface, Kim *et al.* (*23*) have successfully demonstrated various applications, including high-resolution holographic imaging, deep ultraviolet holographic imaging (*24*), 3DHD (*25*), and photon encryption (*26*), highlighting the broad prospects and potential of metasurfaces in the field of holographic display.

In contrast, 3DHDs based on holographic materials use either photopolymers (*27*, *28*), photorefractive materials (*29*), or photochromic materials (*30*) that sense the incident optical field and change their optical properties accordingly to record the hologram. Among these materials, photopolymers have the advantages of high optical sensitivity, high diffraction efficiency, a large dynamic range, and low cost, so they have been widely used in holographic memory applications (*31*). The modulation unit in the photopolymer can be as small as molecular scale, providing a large spatial modulation bandwidth and high spatial resolution in the visualization. Benefitting from this characteristic, researchers have developed high-quality head-mounted displays (*32*, *33*), large-scale 3D displays (*34*, *35*), and real-time 3D displays (*36*). These advanced implementations have mainly focused on increasing the picture size, accelerating the recording speed, and minimizing color distortion, but none of them have improved the fidelity of 3D holography (*37*). The fidelity is mainly constrained by the loss of high-frequency components during light propagation, recording, and reconstruction, which substantially reduces the quality of holographic images and can easily cause eye fatigue during prolonged holographic viewing (*38*). Therefore, finding a way to store and record the high-frequency components in the hologram is important to achieving high-resolution 3D holography.

Here, we present an ultrahigh-fidelity 3D holographic display that uses scattering for homogenization of angular spectrum (HAS). A scattering medium (SM) randomizes the propagation trajectory of the incident photons so that the high-frequency components normally lost during the propagation are randomly remixed with the low-frequency components and then recorded in the hologram with an equal probability. In the display process, the recorded components can be reconstructed by the HAS-3DHD along the original path through the SM. Compared to typical digital devices such as an SLM or DMD, the photopolymer, an analog device with a large number of modulation modes, records scattering holograms at the diffraction limit resolution with a continuous phase modulation (*39*). The proposed HAS-3DHD can more accurately record and recover the angular spectrum distribution after it has been randomized by the SM, thus substantially improving the imaging fidelity of the 3DHD. Because of the spatial multiplexing ability, multiple wavefronts can be sequentially recorded by the photopolymer. In addition, the multiple scattering propagation of light within the SM forms fixed transmission channels, which only allow fields in certain input modes to transmit efficiently (*40*–*42*). This characteristic provides the SM with the ability to select specific transmission channels. Thanks to the spatial multiplexing ability of the photopolymer and the transmission channel selection of the SM, HAS-3DHD can switch between different holograms and realize dynamic, high-resolution 3D holography by precisely adjusting the deflection angle between the photopolymer and the SM. In addition, we introduce a quasi-digital-holography technique that uses a 3D focal scanning module based on a complex amplitude modulation technique as the digital recording source, greatly expanding the recording sources for holographic material-based 3DHD. Our HAS-3DHD demonstrated dynamic 3D holography with a lateral spatial resolution as high as 0.5 μm and a constant contrast ratio of 6 × 10^6^.

## RESULTS

### Principle and characterization of the HAS-3DHD

**Fig. 1.**
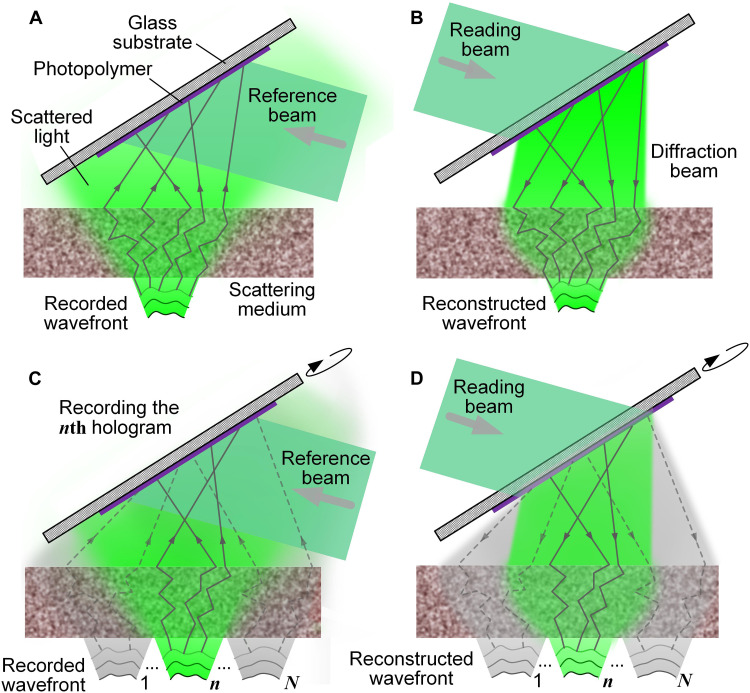
Schematic of HAS-3DHD. (**A**) Recording the wavefront in a photopolymer film behind an SM. (**B**) Reading the hologram in the photopolymer and reconstructing the conjugate wavefront in front of the SM. The reading beam is conjugate to the reference beam. (**C**) Recording multiple wavefronts as holograms at different angles in the photopolymer. The holograms are superimposed over one other. (**D**) Reconstructing these wavefronts sequentially by rotating the photopolymer from α_1_ to α*_N_* while keeping the SM fixed.

Figure 1 is a schematic of HAS-3DHD. The SM fully reorganizes the angular spectrum of the incident light, so that information with different spatial frequencies is randomly distributed across the whole spatial region, and thus the high-frequency components, previously outside of the receiving aperture, can be projected on the photopolymer film. When an introduced reference beam interferes with the scattered optical field, the interference can change the spatial distribution of the refractive index in the photopolymer and create a volume hologram (Fig. 1A). Then, a reading beam coming from the opposite direction of the reference beam propagates through the photopolymer and is shaped by the hologram to a time reversed version of the scattered optical field (Fig. 1B and note S1). This reconstructed light contains the high-frequency components stored in the photopolymer after the angular spectrum homogenization.

For a typical hologram recording, the finite aperture rejects the high-frequency components, limiting the display resolution. In HAS-3DHD, after adding the SM, the incoming photons are randomized, resulting in high-frequency components (which are normally outside the aperture) being redistributed into the aperture. Thus, we can record more high-frequency components in the hologram. Moreover, the photopolymer is an analog device that can record the hologram at the optical diffraction limit resolution. Although the scattering effect is random, it is stable and repeatable. As long as the relative positions of the SM and photopolymer are unchanged, the time reversal of the scattered optical field can propagate backward and reconstruct the conjugate of the incident light before being scattered. Thus, HAS-3DHD can create an ultrahigh-fidelity 3D holographic image.

Although the SM randomizes the incident light, the multiplexing capability of the photopolymer is still preserved since the transmission matrix of the highly SM is linear. We can record multiple holograms in a single piece of photopolymer, and each individual hologram independently records the full information of each scattered light field. When the reading beam irradiates the photopolymer, all the holograms are read out and recreate the superimposed field (note S2). The transmission channel selection capability of the SM enables the photopolymer to support dynamic holography. Let us assume that a dynamic process consists of *N* frames and that we record the wavefront in the *n*th frame (1 < *n* < *N*) after we rotate the photopolymer to an angle α*_n_* (Fig. 1C). In the end, the hologram saved in the photopolymer will be a superposition of all the *N* holograms. When we read the modulation pattern at α*_n_*, only the *n*th wavefront will appear in front of the SM, and all the other wavefronts saved in the other holograms will be scrambled by the SM due to the time reversal symmetry (Fig. 1D and note S4). Owing to this phenomenon, the relative angle between the two media will serve as the basis for selecting the specific transmission channels of the SM, thus filtering out the incorrect hologram recorded at other angles. Therefore, by sequentially rotating the photopolymer from α_1_ to α*_N_*, we can display the whole dynamic process with high resolution and contrast. In comparison, without the SM, all the holograms would be reconstructed and mixed (fig. S1).

**Fig. 2.**
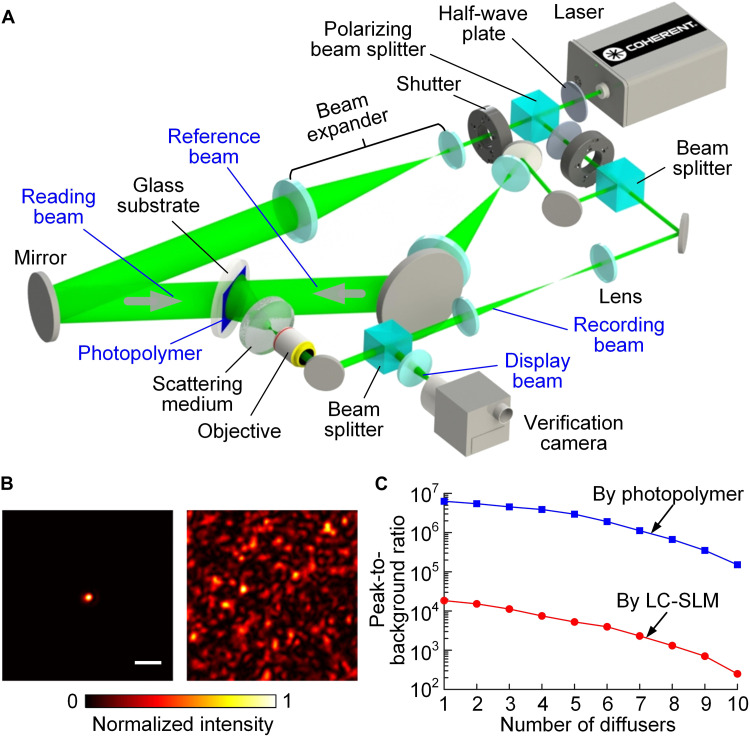
Schematic and characterization of the protype HAS-3DHD. (**A**) Schematic of the experimental setup. (**B**) Normalized intensity distributions corresponding to the reconstructed pattern when the correct (left) and misaligned (right) holograms are loaded into the reading beam. Scale bar, 5 μm. (**C**) Comparison of peak-to-background ratios (PBRs) for different magnitudes of scattering. When the hologram was loaded from the photopolymer (blue line), the reconstructed focus at a particular scattering magnitude had a PBR higher than that generated from digital holography (red line).

We built a prototype holographic system to demonstrate the proposed method (Fig. 2A and Methods). In our demonstration, the reconstructed wavefront corresponded to a focus that was formed in front of the SM by the recording beam passing through an objective with a numerical aperture (NA) of 0.4. The full width at half maximum (FWHM) of the reconstructed focus was ~0.5 μm, very close to the diffraction limit (~0.47 μm) calculated from the NA of the objective (Fig. 2B). This high resolution showed that the photopolymer indeed saved the high-frequency information of the incident wavefront, demonstrating that HAS-3DHD promises ultrahigh-fidelity holography.

The contrast of the reconstructed focus was as high as 6 × 10^6^ due to a combination of factors (Fig. 2B). First, the SM homogenized the angular spectrum of the incident wavefront so that the components across the entire angular spectral range were efficiently used in the reconstruction. Second, the photopolymer has almost two billion modulation modes and can perform continuous phase recording. The pixel size of the photopolymer is on the scale of a half-wavelength, 266 nm in our implementation, which is 30 times smaller than the SLM. As a result, when we replaced the photopolymer with an SLM for loading the hologram, the contrast of the reconstructed focus dropped by a factor of ~300 (Fig. 2C, fig. S2, and note S3). The advantage of HAS-3DHD over the digital method persisted even when we gradually increased the scattering magnitude in the demonstration. Therefore, we demonstrated that the fidelity achieved by HAS-3DHD is much higher than SLM-based digital holographic display.

### Reconstructing a focal spot

**Fig. 3.**
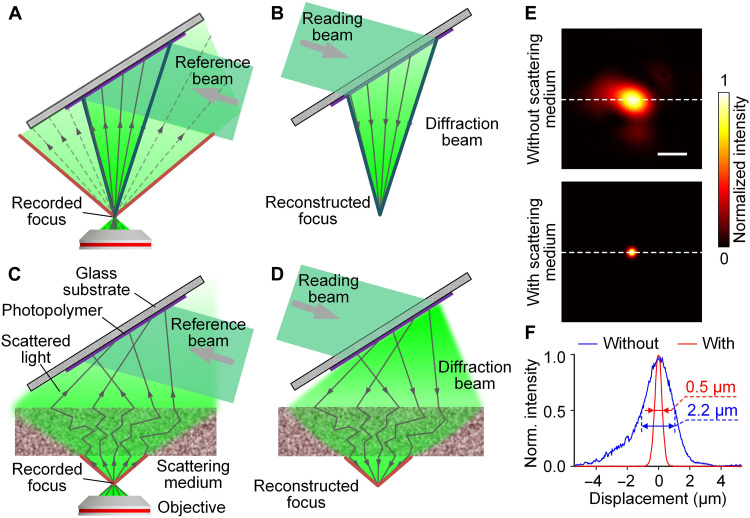
Visualization of angular spectrum homogenization. (**A**) Recording the hologram of focus in the photopolymer through free space. (**B**) Reconstructing the focus without much high-frequency information. (**C**) Recording the hologram of focus through an SM that homogenizes the angular spectrum of the focus. (**D**) Reconstructing the focus with high-frequency information. The red and blue boundaries represent respectively the highest spatial frequency components in the recording beam and in the reconstructed beam through conventional holographic display. (**E**) Normalized intensity distributions of reconstructed foci without (top) and with (bottom) an SM. Scale bar, 2 μm. (**F**) Focal profiles highlighting the improvement in spatial resolution.

To highlight the importance of angular spectrum homogenization, we positioned the photopolymer film far away from the focus. Without an SM, the wavefront focused by a high NA objective diverged quickly. When this diverged wavefront reached the photopolymer, the high-frequency components had propagated outside of the area that the hologram could occupy (Fig. 3A). This part of the information was thus lost in the recording process and could not be recovered in the reading process (Fig. 3B). The loss degraded the spatial resolution of the reconstructed image. Increasing the light-sensitive area of the photopolymer could improve the fidelity of 3DHD to some extent. However, to record all the high-frequency components, the sensing area needs to be extremely large. In addition, the vast majority of holographic image information is concentrated in the low-frequency components. Simply increasing the sensing area would greatly reduce the recording efficiency of the photopolymer. In contrast, if an SM was placed behind the focus, then the propagation path of the incoming photons was randomized, redistributing the angular spectral components. More photons carrying the high-frequency components were redirected by the SM and reached the recording area on the photopolymer film (Fig. 3C). Since many high-frequency components were saved in the hologram, the reading beam could later recover them and display a high-resolution reconstructed image (Fig. 3D). We compared the reconstructed foci without and with an SM (Fig. 3, D and E) when the distance between the focus and the photopolymer was ~20 cm. Without the SM, we observed a large focal spot (FWHM ≈ 2.2 μm) with apparent aberration; with the SM, the focal spot was more tightly focused (FWHM ≈ 0.5 μm). This improvement corresponded to an increase in the acceptance angle from ~7° to ~33°, a result that demonstrates the superior performance of HAS-3DHD.

### Ultrahigh-fidelity holographic image display

**Fig. 4.**
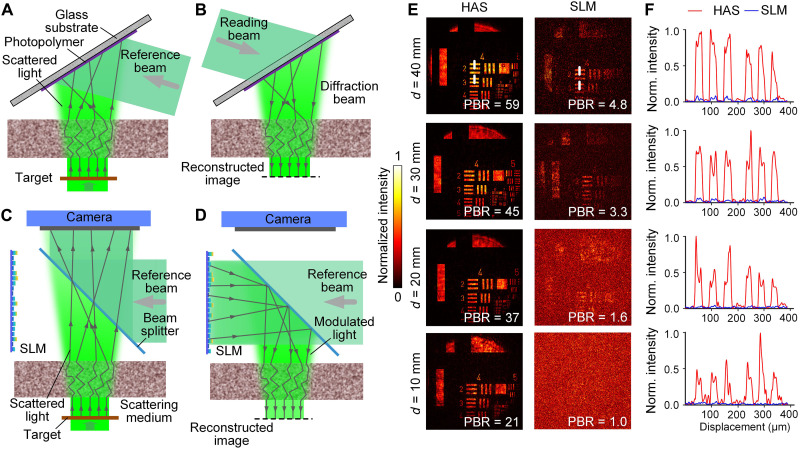
Demonstration of ultrahigh-fidelity image holographic display. (**A** and **B**) Recording and reconstructing a USAF 1951 target through HAS-3DHD. (**C** and **D**) Recording and reconstructing the target through conventional digital holography. (**E**) Comparative holographic displays through HAS-3DHD and SLM-based digital holography at different distances. (**F**) Normalized intensity profiles along the white vertical dashed lines in (E).

To further demonstrate the utility of HAS-3DHD, we characterized its performance with a complex object and compared HAS-3DHD with the SM-enhanced SLM-based 3DHD (*21*). During the recording step, we placed a 1951 United States Air Force (USAF) resolution target in front of the SM (Fig. 4, A and B). When the distance *d* between the target and SM was 40 mm, the peak-to-background ratio (PBR) was 59 (Fig. 4E).

We repeated this experiment with an SLM-based 3DHD, where we put an SLM and camera on the other side of the SM (Fig. 4, C and D, and Methods). The camera recorded the interference pattern between the incident wavefront and the reference beam. A program calculated the phase hologram from the interference and updated the hologram in the SLM, which was conjugated to the camera (fig. S3 and note S4). At the same distance, 40 mm, the virtual image generated by the SLM had a PBR of 4.8 (Fig. 4E), lower than that from HAS-3DHD by a factor of ~12, because of the limited modulation modes and phase discontinuity of the SLM.

This degradation became more prominent when we decreased *d* (fig. S4). As *d* decreased, the speckle size became smaller and the number of speckles increased quadratically. To minimize the loss of information, the holographic system required more modulation modes to control the wavefront of the scattered photons. In this case, the deficiency of the SLM was more obvious. When *d* = 10 mm, the number of modulation modes in the SLM was too few to reconstruct a meaningful image (Fig. 4, E and F). The relationship between PBR and *d* is shown in fig. S4. We tested the quality of the reconstructed resolution target at different wavelengths. The results indicated that the PBRs of the reconstructed images are almost the same at different wavelengths (fig. S11). These results demonstrate that the photopolymer, with a far larger number of modulation modes, could provide display of complex objects with much higher quality than digital methods.

### Dynamic holographic 3D display based on spatially multiplexed holograms

**Fig. 5.**
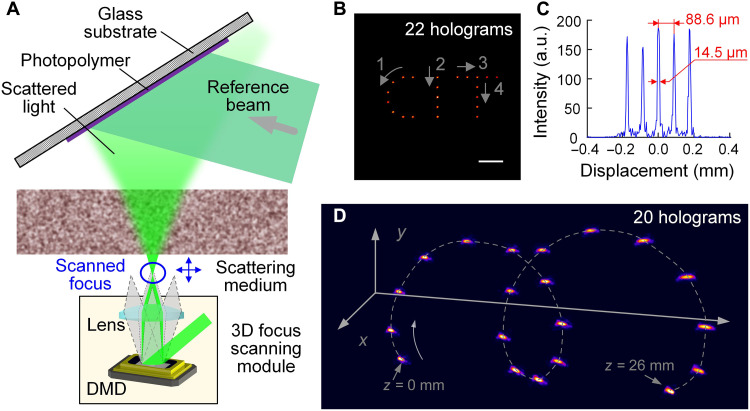
Dynamic 3D display based on HAS-3DHD. (**A**) Illustration of how the dynamic wavefront is generated in the recording step. (**B**) Superposition of frames drawing “CIT” in the image plane. The numbers and arrows illustrate the sequence of frames. Scale bar, 200 μm. (**C**) Intensity profile of the five foci in the letter “I.” (**D**) Superposition of frames drawing a 3D spiral. The curved arrow illustrates the sequence of the frames. a.u., arbitrary units.

We further explored the performance of HAS-3DHD in dynamically displaying complicated graphics in 3D. To generate a focal spot at an arbitrary 3D position, we introduced a flexible digital recording method that used a 3D focus scanning module based on a super-pixel encoded DMD (Fig. 5A and Methods). In the first example, we drew a California Institute of Technology (CIT) pattern with a total of 22 foci at the single image plane. These 22 foci were sequentially produced by the 3D focus scanning module and recorded by the photopolymer after being transmitted through the SM. Then, we reconstructed all the foci at once (Fig. 5B). We analyzed the quality of the focal spot in the letter “I” (Fig. 5C). The intensity variation among focal spots at different locations was less than 8%. We then tried replacing the photopolymer film with an SLM to reconstruct multiple 3D focal points by linearly superimposing multiple holograms, but this proved unfeasible. In the second example, we drew a dynamic 3D spiral (Fig. 5D and movie S1). This dynamic process contained 20 frames corresponding to 20 foci at different 3D positions. The 20 foci were recorded at 20 different orientations of the photopolymer and reconstructed sequentially by precisely rotating the photopolymer. The normalized intensity profiles of the first five foci in the spiral line of the 3D holographic display are shown in fig. S6. The maximum number of frames and points that HAS-3DHD can display depend on the storage capacity of the photopolymer. We used a spatial multiplexing recording technique to consecutively record 92 holograms in the same region of the photopolymer (fig. S8). If the photopolymer is rotated by more than 5 mrad during the recording of each hologram, then a dynamic 3DHD consisting of 92 frames can be generated. Further, holograms can be recorded in different regions of the photopolymer, allowing for a notable increase in the number of frames and points. In practical implementation, this can be achieved by two different ways: translation and rotation (fig. S9). During hologram recording, a specific mask is used to select the current recording region, while the other regions are shielded. Once the recording is complete, the photopolymer can be moved or rotated to the next recording region. This example demonstrates the ability of HAS-3DHD to realize a dynamic holographic display by using the spatial multiplexing capability of the photopolymer and the transmission channel selection capability of the SM so that different images can be displayed independently.

## DISCUSSION

We have developed and demonstrated HAS-3DHD for high-resolution, dynamic 3D holography. HAS-3DHD takes advantage of optical scattering to redistribute image components with different spatial frequencies in the recording beam so that they are projected onto the photopolymer indiscriminately. This process allows recording the high-frequency components that cannot be recorded by conventional holography, enabling an ultrahigh-fidelity holographic display. The lateral spatial resolution reaches the diffraction limit. Because of the limitation of the recording area, the photopolymer film may lose part of the image components during recording. Fortunately, because all frequencies are scattered and redistributed, the composition of the light field frequency domain information is not affected. Therefore, the loss of redistributed components will primarily lead to a decrease in the overall energy of the recorded light field and a corresponding increase of the recording time. Furthermore, because the photopolymer has almost two billion modulation modes, a half optical wavelength scale pixel size (which is 30× smaller than an SLM), and step-free continuous modulation, the contrast of a single focus was improved by a factor of ~300 over that of an SM-enhanced SLM-based 3DHD (*21*). On the basis of the transmission channel selection capability of the SM and the spatial multiplexing capability of the photopolymer, we also demonstrated dynamic 3DHD by sequentially adjusting the deflection angle between the photopolymer and the SM.

HAS-3DHD can be further improved. First, HAS-3DHD is not limited to photopolymers but can be implemented with other various holographic materials. If rewritable holographic materials, such as azobenzene-containing block copolymers (*43*), are used, then HAS-3DHD will be able to realize real-time holographic display. Second, photopolymers and the SM often have a large modulation bandwidth in the visible range, and by integrating with red, green, and blue (RGB) channels and recording each channel at a particular orientation of the photopolymer, HAS-3DHD can be upgraded for full-color holography (*44*–*46*). The display beam with various wavelengths can reconstruct the target at the same position as recorded, minimizing the color distortion. Furthermore, we can improve the photo size of HAS-3DHD by increasing the recording area of the photopolymer film and increase the recording speed by choosing holographic recording materials with shorter response time or using higher laser energy. Because of the scattering effect, the reconstruction efficiency of HAS-3DHD is compromised. Fortunately, it can be improved by enhancing the transmission efficiency of the SM and the diffraction efficiency of the photopolymer. This enhancement can be achieved through strategies such as high-precision system alignment, optimal scattering conditions, and manipulation of incident beams. In addition, by taking advantage of the multiplexing capabilities of holographic materials (*47*), HAS-3DHD can easily switch between different holographic display sources, offering more flexible, dynamic 3D holographic displays.

## METHODS

### Properties of the photopolymer material

We used Bayfol HX 200, a light-sensitive, self-developing photopolymer film, as the holographic material in this work. This film can be used to produce phase holograms in the form of volume reflection and volume transmission holograms within the visible spectral wavelength range from 440 to 680 nm. The material consists of a three-layer stack: a 60 ± 2–μm–thick cellulose triacetate film, a 16 ± 2–μm–thick light-sensitive photopolymer film, and a ~40-μm-thick protective cover film (fig. S12, A and B). When an interfering light field with an energy of more than 30 mJ/cm^2^ irradiates the material, a polymerization reaction begins in the irradiated region. The monomer concentration will decrease at that location, causing a nonuniform distribution of the refractive index within the photopolymer. For hologram formation, no further posttreatment is necessary, neither wet nor thermal.

### Details of the experimental setup

Our holographic system contained four parts that were respectively responsible for recording, reading, reference, and display (Fig. 2A). The 532-nm light from the laser (Verdi G5, Coherent Inc.) was split into two beams by a polarizing beam splitter (PBS051, Thorlabs Inc.). A half-wave plate (WPHSM05-532, Thorlabs Inc.) between the laser and splitter determined the energy division in the two beams by modulating the polarization.

The reflected beam passed through a second half-wave plate that rotated the polarization backward so that the reading beam and reference beam were polarized in the same direction. A pair of shutters excluded interference between the reading and reference beams. After the polarization was rotated back, a beam splitter (BS046, Thorlabs Inc.) again split the beam into a recording beam and a reference beam. The recording beam was then modulated into the incident wavefront by either an objective (MPLN50x, Olympus), a resolution target (R1DS1N, Thorlabs Inc.), or a super-pixel system. The incident wavefront propagated through a stack of three diffusers (DG10-120, Thorlabs Inc.; fig. S12, C and D) to the photopolymer (Bayfol HX200), where it interfered with the reference beam at an angle of 60° to change the distribution of the refractive index in the photopolymer and form the hologram. Driven by a stepper motor, the glass substrate supporting the photopolymer could be rotated to perform dynamic holography.

The reading beam, transmitted through the polarizing beam splitter and a beam expander (20×), was reflected by a mirror to the back side of the photopolymer. This beam, conjugated to the reference beam, was modulated by the photopolymer according to the hologram. The modulated beam propagated back through the diffusers and formed the reconstructed wavefront on the other side. The reconstructed wavefront continued to propagate back along the optical path where the recording beam entered until it encountered a beam splitter that reflected it to a verification camera (PCO.edge 5.5, PCO Corp.).

### Experimental setup of an SLM-based holographic display system

Yu *et al.* (*21*) proposed an SM-enhanced digital holographic display system and realized an ultrahigh-fidelity 3D holographic display by active control of volume speckle fields. We built an experimental system to compare its performance with our HAS-3DHD system (fig. S3). One challenge was that the iterative wavefront shaping algorithms applied by Yu *et al.* (*21*) are extremely slow, taking hours to calculate the phase mask of the SLM. To overcome this challenge, we optimized the system based on digital phase conjugation to calculate the SLM phase mask in milliseconds.

The two shutters, S1 and S2, were open throughout the recording step. The 532-nm light was split into two beams by a polarization beam splitter (PBS051, Thorlabs Inc.). A half-wave plate (WPHSM05-532, Thorlabs Inc.) determined the energy division in the two beams by modulating the polarization. Then, the transmitted beam was expanded by 20× as the reference beam. The reflected beam was used as the recording beam, which was imaged on the object through a 4f system. The recording beam, which carried the 3D information of the object, then passed through the SM and interfered with the reference beam via a beam splitter. The interference field was projected onto the surface of the SLM (HES 6001, Holoeye, 1920 × 1080 pixel count), and the camera (PCO.edge 5.5, PCO, Corp.) was conjugated with the SLM. By adjusting the phase of the reference beam by an electro-optic modulator (350-105, Conoptics, USA), we could calculate the SLM phase mask through the four-step phase shift method and could load it within several milliseconds. After shutter S2 was closed, only the reference beam illuminated the SLM. Once the calculated hologram was loaded, the SLM modulated the reference beam to recover the recorded scattered beam and then displayed the recorded field after passing through the SM.

### 3D focus scanning module

To digitally create a complex wavefront, we built a fast 3D focus scanning module based on a super-pixel encoded DMD (fig. S5). The recording beam was expanded by a 20× beam expander to match the modulation area of the DMD (DLP7000, Texas Instruments). The target field, *E*(*x*, *y*), was converted into a binary pattern to be loaded onto the DMD via a super-pixel encoding look-up table. Once the binary pattern was displayed, the target field was modulated on the focal plane of the 4f system by filtering out the first-order diffracted light through a pinhole. By switching to different binary patterns in order, the holograms could be stored in the photopolymer at a refresh rate of 22.7 kHz.

The 3D focus scanning module controlled the focus 3D position by changing the tilt and defocus coefficient of the light field in front of the focusing lens. The target field, *E*(*x*, *y*), can be expressed by

$$E(x,y)=A\;\exp\left\{\frac{2\pi i}{\lambda }\left[\frac{i}{2(l+f)f}\;(x^{2}+y^{2})+\frac{ax+by}{l+f}\right]\right\}$$

where *f*, *A*, and λ represent the lens focal length, field amplitude, and wavelength, respectively. Further, *a*, *b*, and *l* represent the 3D position of the focal spot. The coordinate origin is the lens focal point. By changing the value of (*a*, *b*, and *l*), the tilt and defocus coefficient of the light field can be changed accordingly, thus focusing the spot at any 3D position.

## Supplementary Materials

### This PDF file includes:

Notes S1 to S5 Figs. S1 to S12 Legend for movie S1 References
