# Supplementary material for: An ultrahigh-fidelity 3D holographic display using scattering to homogenize the angular spectrum
Source: Sci Adv. 2023 Oct 12;9(41):eadi9987. doi: 10.1126/sciadv.adi9987 (PMC10569707; doi:10.1126/sciadv.adi9987)
Supplement: Supplementary file 1 — Notes S1 to S5 Figs. S1 to S12 Legend for movie S1 References [file sciadv.adi9987_sm.pdf]

Science Advances
AAAS

Supplementary Materials for

An ultrahigh-fidelity 3D holographic display using scattering to homogenize the angular spectrum

Jiamiao Yang *et al.*

Corresponding author: Lihong V. Wang, lvw@caltech.edu

Sci. Adv. **9**, eadi9987 (2023)
DOI: 10.1126/sciadv.adi9987

The PDF file includes:

Notes S1 to S5
Figs. S1 to S12
Legend for movie S1
References

Other Supplementary Material for this manuscript includes the following:

Movie S1

Note S1. Theoretical derivation of HAS-3DHD

HAS-3DHD depends on the accurate retrieval of the recorded scattered light field by the photopolymer, otherwise light will not pass through the corresponding transmission channel in the scattering medium (SM) and display the correct holographic image.

Assume the electromagnetic fields of the scattered light and reference beam are

$$E_S(\vec{r}) = E_S \cdot \exp\left(j\vec{k}_S \cdot \vec{r}\right),$$
$$E_R(\vec{r}) = E_R \cdot \exp\left(j\vec{k}_R \cdot \vec{r}\right). \tag{S1}$$

From the perspective of the transmission matrix, the scattered light $E_S = T \cdot E_{\text{rec}}$, where E_{rec} is the recorded field carrying the object information and *T* is the transmission matrix of the SM, which consists of $M \times N$ complex coefficients k_{mn} connecting the *n*-th node in the recorded field and the *m*-th node in the scattered field $(1 \leq m \leq M,\ 1 \leq n \leq N)$. k_{mn} follows a circular Gaussian distribution. E_S and the reference field E_R interfere and form a hologram on the photopolymer. Then E_S is recovered from the hologram under irradiation by the reading beam. If the relative positions of the SM and photopolymer remain unchanged, then E_{rec} will be retrieved because $E_{\text{rec}} = T^{-1} \cdot E_S$.

The interference intensity can be written as

$$I_{\text{holo}}(\vec{r}) = |E_S|^2 + |E_R|^2 + 2E_S E_R \cos\left[\left(\vec{k}_R - \vec{k}_S\right) \cdot \vec{r}\right]. \tag{S2}$$

The photopolymer is illuminated by the interference pattern and records a holographic grating with amplitude and phase modulation. Its transmission function can be represented as

$$t(\vec{r}) = \left[t_0 + t_1 \cos\left(\vec{K} \cdot \vec{r}\right)\right] \cdot \exp\left\{j\left[\varphi_0 + \varphi_1 \cos\left(\vec{K} \cdot \vec{r}\right)\right]\right\}, \tag{S3}$$

where $\vec{K} = \vec{k}_R - \vec{k}_S$ is the grating vector, t_0 is the background transmission, t_1 is the peak amplitude-related transmission modulation rate, φ_0 is the average phase change through this material, and φ_1 is the peak phase modulation.

When using the conjugated reference beam to read out the recorded hologram, the optical field of playback beam is:

$$
\begin{aligned}
E_{\text{playback}}(\vec{r}) &= E_R^*(\vec{r}) \cdot t(\vec{r}) \\
&= E_R \exp\left(-j\vec{k}_R \cdot \vec{r}\right) \cdot \left[t_0 + t_1 \cos\left(\vec{K} \cdot \vec{r}\right)\right] \\
&\quad \cdot \exp\left\{j\left[\varphi_0 + \varphi_1 \cos\left(\vec{K} \cdot \vec{r}\right)\right]\right\} \\
&= E_R \exp(j\varphi_0) \exp(j\varphi_0) \cdot \exp\left(-j\vec{k}_R \cdot \vec{r}\right) \cdot \left[t_0 + t_1 \cos\left(\vec{K} \cdot \vec{r}\right)\right] \\
&\quad \cdot \exp\left[j\varphi_1 \cos\left(\vec{K} \cdot \vec{r}\right)\right] \\
&\approx E_R t_0 \exp(j\varphi_0) \cdot \exp\left(-j\vec{k}_R \cdot \vec{r}\right) \exp\left[j\varphi_1 \cos\left(\vec{K} \cdot \vec{r}\right)\right].
\end{aligned} \tag{S4}
$$

According to the Jacobi–Anger expansion,

$$
\exp\left[j\varphi_1 \cos\left(\vec{K} \cdot \vec{r}\right)\right] = \sum_{N=-\infty}^{\infty} j^N J_N(\varphi_1) \exp\left[jN\vec{K} \cdot \vec{r}\right]. \tag{S5}
$$

We only consider $N = 0$ and 1,

$$
\exp\left[j\varphi_1 \cos\left(\vec{K} \cdot \vec{r}\right)\right] = J_0(\varphi_1) + jJ_1(\varphi_1) \exp\left(j\vec{K} \cdot \vec{r}\right). \tag{S6}
$$

Thus,

$$
\begin{aligned}
E_{\text{playback}}(\vec{r}) &= jE_R t_0 \exp(j\varphi_0) \cdot \exp\left(-j\vec{k}_R \cdot \vec{r}\right) \left[J_0(\varphi_1) + J_1(\varphi_1) \exp\left(j\vec{K} \cdot \vec{r}\right)\right] \\
&= jE_R t_0 J_1(\varphi_1) \exp(j\varphi_0) \exp\left(-j\vec{k}_S \cdot \vec{r}\right) + jE_R t_0 J_0(\varphi_1) \exp(j\varphi_0) \\
&\quad \cdot \exp\left(-j\vec{k}_R \cdot \vec{r}\right).
\end{aligned} \tag{S7}
$$

Thus, the first item on the right side gives the conjugated phase of the scattered field E_S, i.e., the recorded field E_{rec} is also recovered.

Note S2. Recording and recovering multiply scattered holograms

When the SM and photopolymer remain motionless, if multiple recorded light fields carrying different object information interfere one by one with the reference light field, they will form a superimposed hologram on the photopolymer. Under irradiation, all the recorded light fields will be recovered at once, and the superimposed images of multiple objects will be displayed. The theoretical proof is as follows.

The sum of the holograms' intensities is

$$
\sum_{m=1}^{M} I_{\text{holo}}(\vec{r}) = \sum_{m=1}^{M} |E_{Sm}|^2 + M|E_R|^2 + 2E_R \sum_{m=1}^{M} E_{Sm} \cos\left[\left(\vec{k}_R - \vec{k}_{Sm}\right) \cdot \vec{r}\right]. \tag{S8}
$$

Then

$$t(\vec{r})=t_0\exp(j\varphi_0)\exp\left\{j2E_R\sum_{m=1}^{M}E_{Sm}\cos\left[\left(\vec{k}_R-\vec{k}_{Sm}\right)\cdot\vec{r}\right]\right\}$$
$$=t_0\exp(j\varphi_0)\prod_{m=1}^{M}\exp\left\{j\varphi_m\cos\left[\left(\vec{k}_R-\vec{k}_{Sm}\right)\cdot\vec{r}\right]\right\}, \quad \text{(S9)}$$

where $\varphi_m=2E_R E_{Sm}$.

According to the Jacobi–Anger expansion,

$$\exp\left\{j\varphi_m\cos\left[\left(\vec{k}_R-\vec{k}_{Sm}\right)\cdot\vec{r}\right]\right\}=\sum_{N=-\infty}^{\infty}j^N J_N(\varphi_m)\exp\left[jN\left(\vec{k}_R-\vec{k}_{Sm}\right)\cdot\vec{r}\right]. \text{(S10)}$$

Only consider $N = 0$ and 1

$$\exp\left\{j\varphi_m\cos\left[\left(\vec{k}_R-\vec{k}_{Sm}\right)\cdot\vec{r}\right]\right\}=J_0(\varphi_m)+jJ_1(\varphi_m)\exp\left[j\left(\vec{k}_R-\vec{k}_{Sm}\right)\cdot\vec{r}\right].$$

(S11)

Thus,

$$t(\vec{r})=t_0\exp(j\varphi_0)\prod_{m=1}^{M}\left\{J_0(\varphi_m)+jJ_1(\varphi_m)\exp\left[j\left(\vec{k}_R-\vec{k}_{Sm}\right)\cdot\vec{r}\right]\right\}, \quad \text{(S12)}$$

and then

$$E_{\text{playback}}(\vec{r})=E_R^*(\vec{r})\cdot t(\vec{r})$$
$$=E_R t_0\exp(j\varphi_0)\exp\left(-j\vec{k}_R\cdot\vec{r}\right)$$
$$\cdot\prod_{m=1}^{M}\left\{J_0(\varphi_m)+jJ_1(\varphi_m)\exp\left[j\left(\vec{k}_R-\vec{k}_{Sm}\right)\cdot\vec{r}\right]\right\}. \quad \text{(S13)}$$

Thus we always can find a term of $\exp\left(-j\vec{k}_R\cdot\vec{r}\right)\exp\left[j\left(\vec{k}_R-\vec{k}_{Sm}\right)\cdot\vec{r}\right]$ to playback a conjugated scattered light with a phase of $\exp\left(-j\vec{k}_{Sm}\cdot\vec{r}\right)$.

Note S3. The theoretical maximum peak-to-background ratio (PBR) of focus through scattering medium modulated by the phase-only modulation devices

For simplicity, we assume that each element in the transmission matrix T is drawn from a circular gaussian distribution with mean $\mu=0$ and $\sigma_{\text{real}}=\sigma_{\text{complex}}=\sigma$. In the cases when T is a unitary matrix such that $TT^{\dagger}=I$, σ can be proved to be $1/\sqrt{2N}$, where N is the dimension of the matrix.

We assume the input field is E_1^A, with only the first element to be nonzero while the rest elements are zero. Also, both photopolymer and LC-SLM have phase only modulation capability. Thus, the peak of the playback field is calculated as

$$E_1^{A'} = E_1^A \sum_{n=1}^{N} T_{1n}^{BA} \exp(-i\angle T_{n1}^{AB}) = E_1^A \sum_{n=1}^{N} |T_{1n}^{BA}|. \tag{S14}$$

When N is large, the discrete summation can be converted into continuous integration.

$$\begin{aligned} \sum_{N=1}^{\infty} |T_{1n}^{BA}| &= N \int_{-\infty}^{+\infty} \int_{-\infty}^{+\infty} \sqrt{x^2+y^2} f(x) f(y) dxdy, \\ &= N \int_0^{2\pi} \int_0^{+\infty} \frac{1}{2\pi\sigma^2} \exp(-r^2/2\sigma^2) r^2 dr d\theta, \\ &= N \int_0^{+\infty} \frac{1}{2\sigma^2} \exp\left(-\left(r/\sqrt{2}\,\sigma\right)^2\right) r d(r^2), \\ &= N\sqrt{2}\,\sigma\Gamma(3/2) \\ &= \frac{\sqrt{2}}{2} \sigma \sqrt{\pi} N. \end{aligned} \tag{S15}$$

Thus,

$$I_1^{A'} = \frac{1}{2} \sigma^2 \pi N^2 I_1^A. \tag{S16}$$

Then, the background of the playback field is calculated as

$$E_{m\neq 1}^{A'} = E_1^A \sum_{n=1}^{N} T_{mn}^{BA} \exp(-j\angle T_{n1}^{AB}). \tag{S17}$$

Thus, the background intensity is

$$I_{m\neq 1}^{A'} = I_1^A < |\sum_{n=1}^{N} T_{mn}^{BA} \exp(-j\angle T_{n1}^{AB})|^2 > \tag{S18}$$

Here, $<\cdots>$ denotes the ensemble average. Since T_{mn}^{BA} satisfy a circular gaussian distribution and $\exp(-j\angle T_{n1}^{AB})$ has a randomly distributed phase, their product $T_{mn}^{BA} \exp(-j\angle T_{n1}^{AB})$ also satisfies the same circular gaussian distribution. Thus, we get

$$I_{m\neq 1}^{A'} = I_1^A N 2\sigma^2. \tag{S19}$$

Finally, we can calculate the peak-background ratio (PBR) of the focus

$$\mathrm{PBR} = \frac{I_1^{A'}}{I_{m\neq 1}^{A'}} = \frac{\frac{1}{2}\sigma^2 \pi N^2}{2N\sigma^2} = \frac{\pi}{4} N. \tag{S20}$$

The PBR of the recovered focus is proportional to the modulation modes for a holographic device with phase-only modulation. Moreover, the modulation modes of LC-SLM is limited (commonly 2 million modes for commercial high-resolution SLM), thus the PBR of focus reconstructed by the LC-SLM is limited by the modulation modes (Fig. S2). In contrast, since the photopolymer is an analog device, the modulation unit of the photopolymer is much smaller than the wavelength-level speckles. The modulation modes of the photopolymer is more than two orders of magnitude higher than LC-SLM, hence the PBR of the focus is much higher, which is consistent with the results in Fig.1c.

Note S4. Dynamic 3D holographic display enabled by the memory effect

$$C(\Delta x, \Delta \mathbf{k}) = \exp\left(-\frac{L^3 k_0^2}{2l_{tr}}\left[\frac{|\Delta \mathbf{k}|^2}{3k_0^2} - \frac{\Delta \mathbf{k}\cdot\Delta x}{k_0 L} + \frac{|\Delta x|^2}{L^2}\right]\right), \tag{S21}$$

where k_0, L, and l_{tr} are the wavenumber, diffusion thickness, and transport mean free path, respectively.

In dynamic holography, we consider that the deflection $\Delta \boldsymbol{k}$ between the beam and the SM remains the same, and only the shift Δx changes, so

$$C(\Delta x) = \exp\left(-\frac{Lk_0^2}{2l_{tr}}|\Delta x|^2\right). \tag{S22}$$

We define $\Delta\phi = x/r$, $0 \leq r \leq R$, where R is the beam radius, and then the autocorrelation coefficient of the transmission matrix before and after beam rotation is

$$C(\Delta\phi) = \frac{1}{\pi R^2}\int_0^{2\pi}\int_0^R \exp\left(-\frac{Lk_0^2}{2l_{tr}}|r\,\Delta\phi|^2\right) r\,\mathrm{d}r\,\mathrm{d}\theta. \tag{S23}$$

We performed a simple numerical simulation of the memory effect. When L, l_{tr}, R, and the wavelength have values of 258 μm, 14.8 mm which was same as Reference (*48*), 5 mm, and 532 nm, respectively, the relationship between $C(\Delta\phi)$ and $\Delta\phi$ is as depicted in Fig. S7. That is, when the rotation angle $\Delta\phi$ deviates by over 1 mrad, the transmission matrix can be considered as irrelevant. Thus, we can display the correct frame image. In our implementation, we utilized the 3D focus scanning module shown in Fig. S5 to generate two focused spots at different locations. The photopolymer was rotated over ~5 mrad using an electric precision rotary stage, and the positions of both foci were recorded with the HAS-3DHD prototype. During the reconstruction, we captured the intensity distribution of each focus separately with an sCMOS camera (Fig. S10a and b). Fig. S10c displays the lateral intensity profiles of the two foci. It can be observed that after rotating the photopolymer over 5 mrad, the adjacent images could be completely separated.

Note S5. The relationship between the PBR and distance *d* from the holographic target and scattering medium

The peak-to-background ratio (PBR) of the reconstructed focus in HAS-3DHD is proportional to the ratio of the number of control modes *N* in the photopolymer to the number of speckles in the reconstructed image *M* (*49*, *50*). In particular, as the distance *d* between the recorded object (USAF 1951 resolution chart) and the scattering medium increases, the diameter of the speckles D_s will also increase, following the relationship (*51*)

$$D_s = \frac{0.61\sqrt{\pi}\,\lambda d}{\sqrt{A}} \tag{S24}$$

Here, λ represents light wavelength, A represents the total illuminated area of the SM. Therefore, the number of speckles in the reconstructed image M decreases as the distance d increases, which means the PBR increases with increasing d. The relationship between PBR and d can be theoretically expressed as follows:

$$\begin{aligned} \mathrm{PBR} &= K\frac{N}{M} \\ &= K\frac{N}{S/D_s^2} = K\frac{N}{S}D_s^2 \\ &= K'\frac{N\lambda^2}{SA}d^2 \end{aligned} \tag{S25}$$

Here, D_s represents speckle size at the recorded and reconstructed position; S represents the effected modulation area; K represents a constant related to the modulation capability of the photopolymer for phase modulation, $K=\pi/4\,;K'=0.61^2\pi K$. In practical scenarios, due to the influence of various noise factors, the above relationship may not strictly hold as d increases.

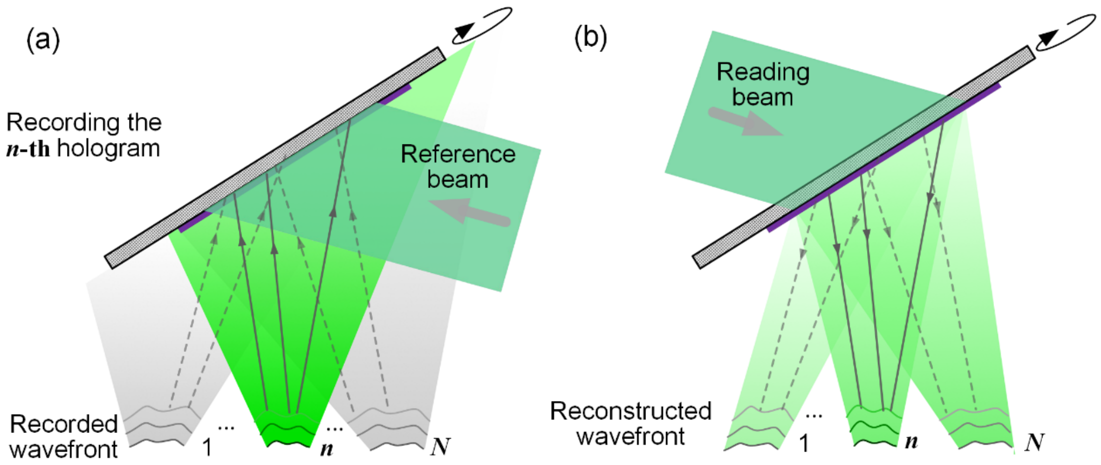

Fig. S1. Principle of spatial multiplexing with a photopolymer. **(a)** Multiple wavefronts are sequentially recorded as holograms on the photopolymer. The holograms are superimposed over each other at the same position. **(b)** In the absence of scattering medium transmission channel selection, all the recorded wavefronts are reconstructed at the same time, therefore dynamic display cannot be achieved.

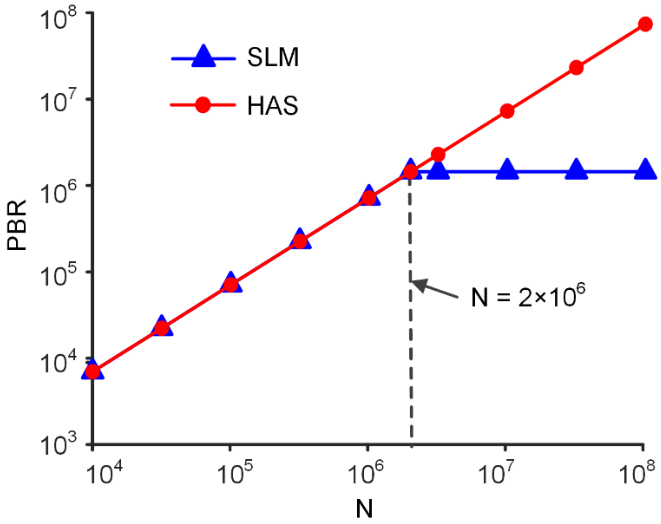

Fig. S2. PBRs of a reconstructed focus, using the HAS-3DHD or SLM-based holographic display with different modulation modes, N.

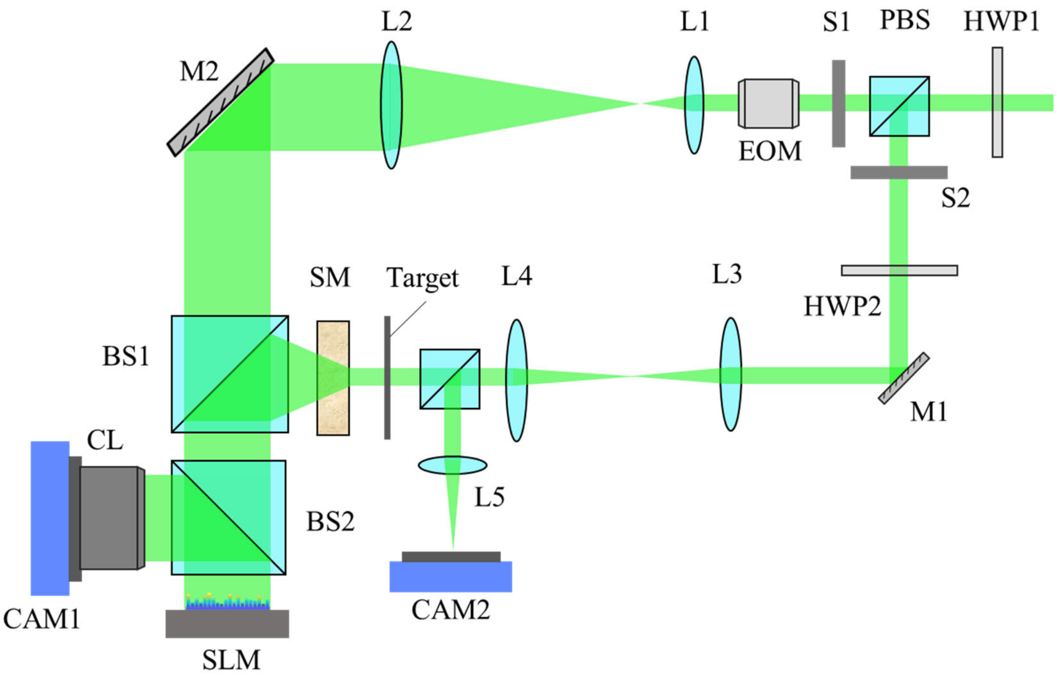

Fig. S3. Experimental set-up of the scattered spatial light modulator (SLM)-based holographic display prototype. HWP, half-wave plate; S, shutter; M, mirror; L, lens; BS, beam splitter; PBS, polarized beam splitter; SM, scattering medium; CAM, camera; CL, camera lens; EOM, electro-optic modulator.

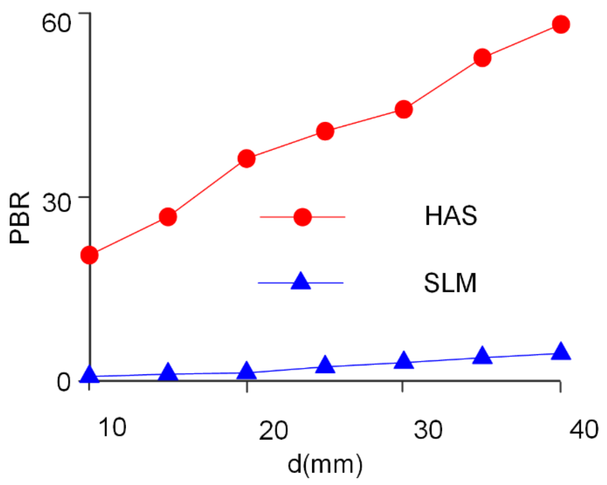

Fig. S4. PBRs of a reconstructed image of a 1951USAF test target, using the HAS-3DHD or SLM-based holographic display at different distances, d.

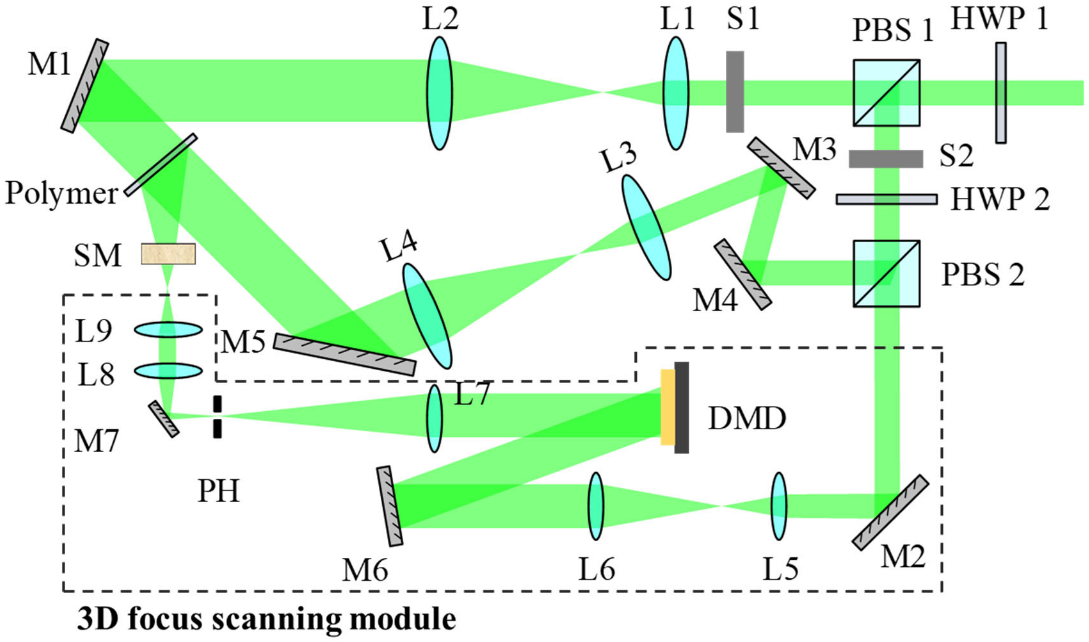

Fig. S5. Hologram recording system based on a 3D focus scanning module. DMD, digital micromirror; PH, pinhole.

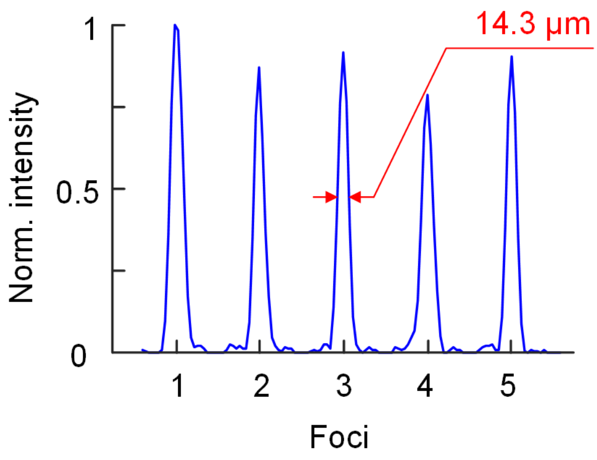

Fig. S6. Normalized intensity profile of the first five foci in the spiral line 3D holographic display.

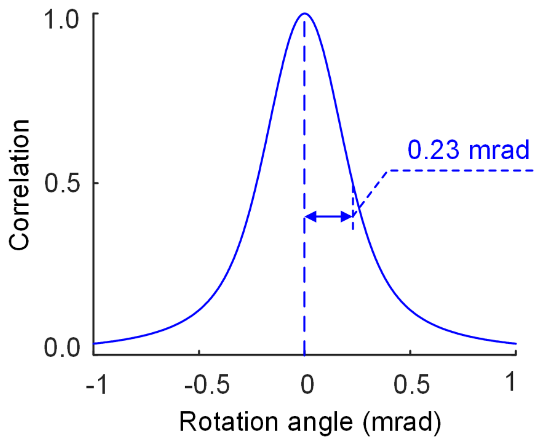

Fig. S7. The correlation function between the transmission matrix and different rotation angle.

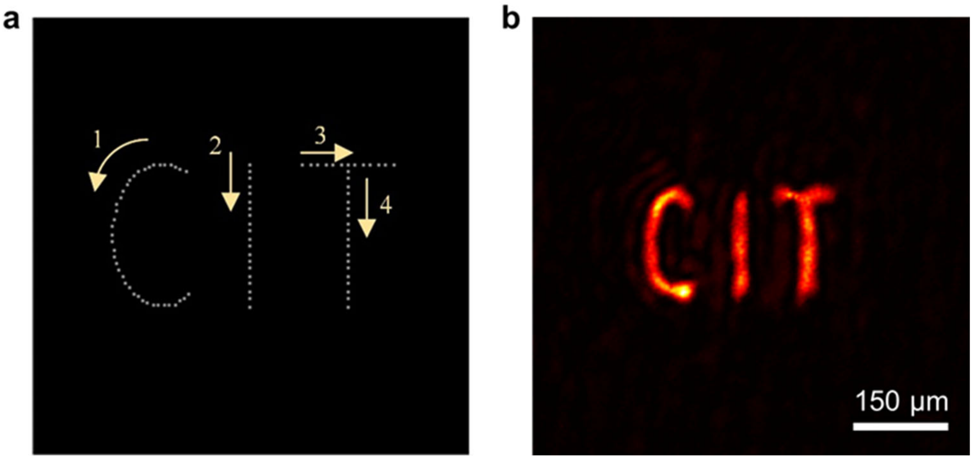

Fig. S8. Increasing the number of points by recording multiple holograms. (**a**) Scanning path of the 3D focus scanning module. The numbers and arrows illustrate the sequence of 92 frames. (**b**) Superposition of frames drawing "CIT" in the image plane.

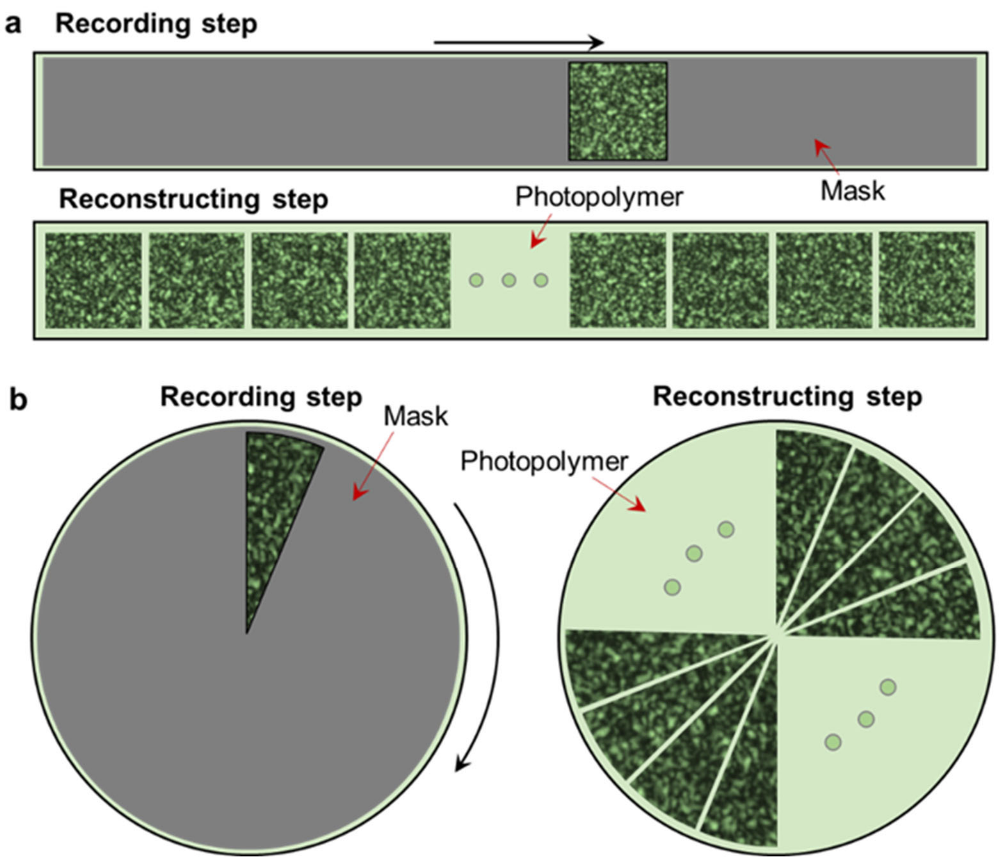

Fig. S9. Increasing the number of points by recording holograms in different areas of the photopolymer. (**a**) Translational spatial multiplexing. (**b**) Rotating spatial multiplexing. The black arrow indicates the direction photopolymer moves or rotates. Masks are added to block stray light in the recording step.

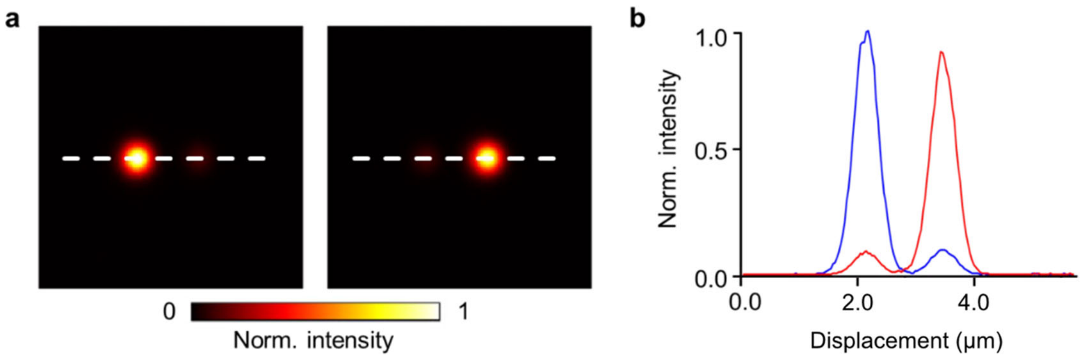

Fig. S10. Reconstructing adjacent images utilizing the memory effect of scattering medium and the multiplexing ability of photopolymer. (**a**) Reconstructing the foci separately by rotating the photopolymer by 5 mrad. (**b**) Normalized intensity profiles along the vertical white dashed lines in (a).

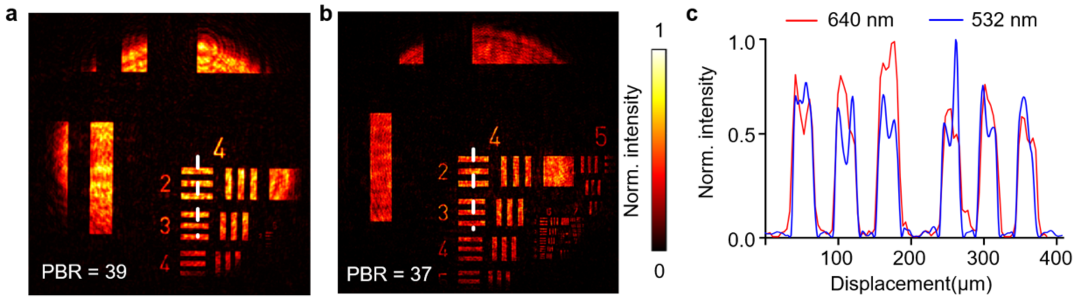

Fig. S11. Reconstructing the holographic image at different wavelengths. (**a**, **b**) The reconstructed resolution target when the distance d between the target and SM was 20 mm at 640 nm (a) and 532 nm (b). (**c**) Normalized intensity profiles along the vertical white dashed lines in (a) at 640 nm and 532 nm.

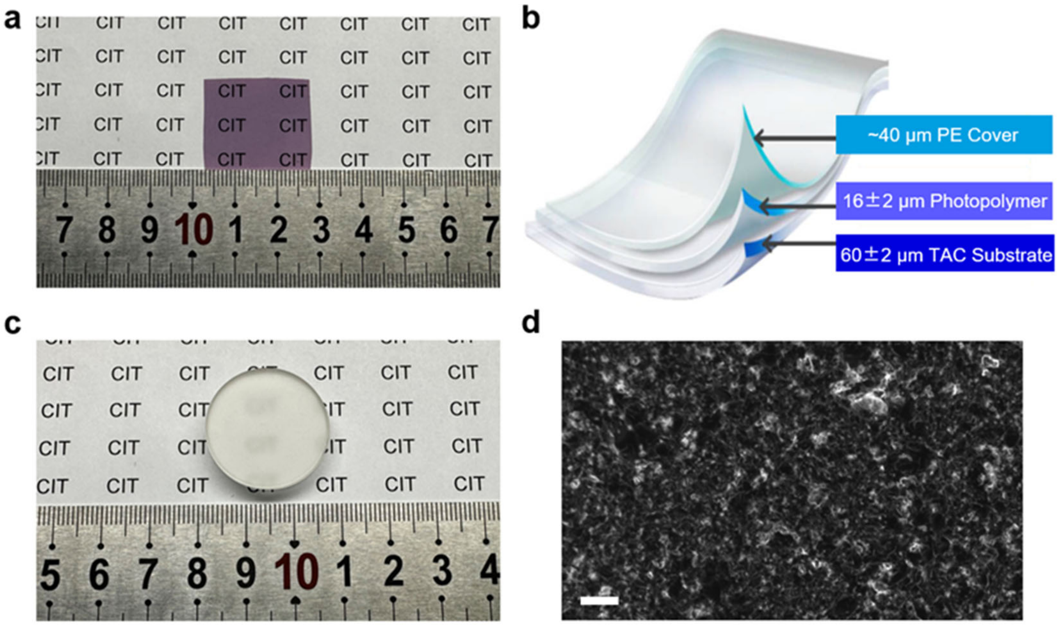

Fig. S12. The images of the photopolymer and diffuser used in the HAS-3DHD experiments. (**a**) A photographic image of the photopolymer. (**b**) A schematic of the photopolymer structure, comprising a three-layer stack: a 60±2 µm thick cellulose (TAC) substrate, a 16 ± 2 µm thick light-sensitive photopolymer film, and a ~40 µm thick protective PE cover film, which is removed prior to hologram recording (*52*). (**c**) A photographic image of the diffuser. (**d**) A microscope image of the diffuser. Scale bar, 250 µm.

Movie S1. The dynamic 3D spiral holographic display result. This 3D spiral contained 20 frames corresponding to 20 foci at different 3D positions. The foci position was controlled by the 3D focus scanning module. The 20 foci were recorded at 20 different orientations of the photopolymer and reconstructed sequentially by precisely rotating the photopolymer.

REFERENCES AND NOTES

1. D. Pi, J. Liu, Y. Wang, Review of computer-generated hologram algorithms for color dynamic holographic three-dimensional display. *Light Sci. Appl.* **11**, 231 (2022).

2. Y. Pan, J. Liu, X. Li, Y. Wang, A review of dynamic holographic three-dimensional display: Algorithms, devices, and systems. *IEEE Trans. Industr. Inform.* **12**, 1599–1610 (2015).

3. H. Gao, Y. Wang, X. Fan, B. Jiao, T. Li, C. Shang, C. Zeng, L. Deng, W. Xiong, J. Xia, M. Hong, Dynamic 3D meta-holography in visible range with large frame number and high frame rate. *Sci. Adv.* **6**, eaba8595 (2020).

4. G. Li, D. Lee, Y. Jeong, J. Cho, B. Lee, Holographic display for see-through augmented reality using mirror-lens holographic optical element. *Opt. Lett.* **41**, 2486–2489 (2016).

5 D. Gabor, Holography, 1948-1971. *Science* **177**, 299–313 (1972).

6. Z. He, X. Sui, G. Jin, L. Cao, Progress in virtual reality and augmented reality based on holographic display. *Appl. Opt.* **58**, A74–A81 (2019).

7. T. Kreis, Application of digital holography for nondestructive testing and metrology: A review. *IEEE Trans. Industr. Inform.* **12**, 240–247 (2015).

8. D. Mavrikios, K. Alexopoulos, K. Georgoulias, S. Makris, G. Michalos, G. Chryssolouris, Using Holograms for visualizing and interacting with educational content in a Teaching Factory. *Procedia Manuf.* **31**: 404–410 (2019).

9. Y. Shi, C. Wan, C. Dai, Z. Wang, S. Wan, G. Zheng, S. Zhang, Z. Li, Augmented reality enabled by on-chip meta-holography multiplexing. *Laser Photonics Rev.* **16**, 2100638 (2022).

10. V. A. Barachevsky, The current status of the development of light-sensitive media for holography (a review). *Opt. Spectrosc.* **124**, 373–407 (2018).

11. P. Zhou, Y. Li, X. Li, S. Liu, Y. Su, Holographic display and storage based on photo-responsive liquid crystals. *Liq. Cryst. Rev.* **4**, 83–100 (2016).

12. S. Jiao, Z. Zhuang, W. Zou, Fast computer generated hologram calculation with a mini look-up table incorporated with radial symmetric interpolation. *Opt. Express* **25**, 112–123 (2017).

13. S. De Nicola, A. Finizio, G. Pierattini, P. Ferraro, D. Alfieri, Angular spectrum method with correction of anamorphism for numerical reconstruction of digital holograms on tilted planes. *Opt. Express* **13**, 9935–9940 (2005).

14. T. Tommasi, B. Bianco, Computer-generated holograms of tilted planes by a spatial frequency approach. *J. Opt. Soc. Am. A* **10**, 299–305 (1993).

15. M. H. Eybposh, N. W. Caira, M. Atisa, P. Chakravarthula, N. C. Pégard, DeepCGH: 3D computer-generated holography using deep learning. *Opt. Express* **28**, 26636–26650 (2020).

16. R. Horisaki, R. Takagi, J. Tanida, Deep-learning-generated holography. *Appl. Opt.* **57**, 3859–3863 (2018).

17. J. Cho, S. Kim, S. Park, B. Lee, H. Kim, DC-free on-axis holographic display using a phase-only spatial light modulator. *Opt. Lett.* **43**, 3397–3400 (2018).

18. T. Kozacki, Holographic display with tilted spatial light modulator. *Appl. Opt.* **50**, 3579–3588 (2011).

19. M. C. Park, B. R. Lee, J. Y. Son, O. Chernyshov, Properties of DMDs for holographic displays. *J. Mod. Opt.* **62**, 1600–1607 (2015).

20. M. Agour, C. Falldorf, R. B. Bergmann, Holographic display system for dynamic synthesis of 3D light fields with increased space bandwidth product. *Opt. Express* **24**, 14393–14405 (2016).

21. H. Yu, K. Lee, J. Park, Y. Park, Ultrahigh-definition dynamic 3D holographic display by active control of volume speckle fields. *Nat. Photonics* **11**, 186–192 (2017).

22. J. Kim, J. Seong, W. Kim, G.-Y. Lee, S. Kim, H. Kim, S.-W. Moon, D. K. Oh, Y. Yang, J. Park, J. Jang, Y. Kim, M. Jeong, C. Park, H. Choi, G. Jeon, K.-I. Lee, D. H. Yoon, N. Park, B.

Lee, H. Lee, J. Rho, Scalable manufacturing of high-index atomic layer–polymer hybrid metasurfaces for metaphotonics in the visible. *Nat. Mater.* **22**, 474–481 (2023).

23. J. Kim, D. K. Oh, H. Kim, G. Yoon, C. Jung, J. K. Kim, T. Badloe, S. Kim, Y. Yang, J. Lee, B. Ko, J. G. Ok, J. Rho, Metasurface holography reaching the highest efficiency limit in the visible via one-step nanoparticle-embedded-resin printing. *Laser Photonics Rev.* **16**, 2200098 (2022).

24. J. Kim, W. Kim, D. K. Oh, H. Kang, H. Kim, T. Badloe, S. Kim, C. Park, H. Choi, H. Lee, J. Rho, One-step printable platform for high-efficiency metasurfaces down to the deep-ultraviolet region. *Light Sci. Appl.* **12**, 68 (2023).

25. S. So, J. Kim, T. Badloe, C. Lee, Y. Yang, H. Kang, J. Rho, Multicolor and 3D Holography Generated by Inverse-Designed Single-Cell Metasurfaces. *Adv. Mater.* **35**, e2208520 (2023).

26. J. Kim, D. Jeon, J. Seong, T. Badloe, N. Jeon, G. Kim, J. Kim, S. Baek, J.-L. Lee, J. Rho, Photonic encryption platform *via* dual-band vectorial metaholograms in the ultraviolet and visible. *ACS Nano* **16**, 3546–3553 (2022).

27. N. Ishii, T. Kato, J. Abe, A real-time dynamic holographic material using a fast photochromic molecule. *Sci. Rep.* **2**, 819 (2012).

28. T. J. Trentler, J. E. Boyd, V. L. Colvin, Epoxy resin−photopolymer composites for volume holography. *Chem. Mater.* **12**, 1431–1438 (2000).

29. P. Günter, Holography, coherent light amplification and optical phase conjugation with photorefractive materials. *Phys. Rep.* **93**, 199–299 (1982).

30. Y. Kobayashi, J. Abe, Real-Time Dynamic Hologram of a 3D Object with Fast Photochromic Molecules. *Adv. Opt. Mater.* **4**, 1354–1357 (2016).

31. J. Guo, M. R. Gleeson, J. T. Sheridan, A review of the optimisation of photopolymer materials for holographic data storage. *Phys. Res. Int.* **2012**, 803439 (2012).

32. J.-A. Piao, G. Li, M.-L. Piao, N. Kim, Full color holographic optical element fabrication for waveguide-type head mounted display using photopolymer. *J. Opt. Soc. Korea* **17**, 242–248 (2013).

33. S. Lee, B. Lee, J. Cho, C. Jang, J. Kim, B. Lee, Analysis and implementation of hologram lenses for see-through head-mounted display. *IEEE Photon. Technol. Lett.* **29**, 82–85 (2016).

34. P. A. Blanche, A. Bablumian, R. Voorakaranam, C. Christenson, W. Lin, T. Gu, D. Flores, P. Wang, W. Y. Hsieh, M. Kathaperumal, B. Rachwal, O. Siddiqui, J. Thomas, R. A. Norwood, M. Yamamoto, N. Peyghambarian, Holographic three-dimensional telepresence using large-area photorefractive polymer. *Nature* **468**, 80–83 (2010).

35. S. H. Lin, K. Y. Hsu, W. Z. Chen, W. T. Whang, Phenanthrenequinone-doped poly (methyl methacrylate) photopolymer bulk for volume holographic data storage. *Opt. Lett.* **25**, 451–453 (2000).

36. J. Zhu, G. Wang, Y. Hao, B. Xie, A. Y. S. Cheng, Highly sensitive and spatially resolved polyvinyl alcohol/acrylamide photopolymer for real-time holographic applications. *Opt. Express* **18**, 18106–18112 (2010).

37. S. Tay, P. A. Blanche, R. Voorakaranam, A. V. Tunç, W. Lin, S. Rokutanda, T. Gu, D. Flores, P. Wang, G. Li, P. St Hilaire, J. Thomas, R. A. Norwood, M. Yamamoto, N. Peyghambarian, An updatable holographic three-dimensional display. *Nature* **451**, 694–698 (2008).

38. M. Rosenfield, Computer vision syndrome: A review of ocular causes and potential treatments. *Ophthalmic Physiol. Opt.* **31**, 502–515 (2011).

39. H. Lai, X. Peng, L. Li, D. Zhu, P. Xiao, Novel monomers for photopolymer networks. *Prog. Polym. Sci.* **128**, 101529 (2022).

40. H. Yılmaz, M. Kühmayer, C. W. Hsu, S. Rotter, H. Cao, Customizing the angular memory effect for scattering media, *Phys. Rev. X* **11**, 031010 (2021).

41. S. Yoon, M. Kim, M. Jang, Y. Choi, W. Choi, S. Kang, W. Choi, Deep optical imaging within complex scattering media. *Nat. Rev. Phys.* **2**, 141–158 (2020).

42. A. K. Singh, D. N. Naik, G. Pedrini, M. Takeda, W. Osten, Exploiting scattering media for exploring 3D objects. *Light Sci. Appl.* **6**, e16219 (2017).

43. M. Häckel, L. Kador, D. Kropp, H. W. Schmidt, Polymer Blends with Azobenzene-Containing Block Copolymers as Stable Rewritable Volume Holographic Media. *Adv. Mater.* **19**, 227–231 (2007).

44. M. Ozaki, J. Kato, S. Kawata, Surface-plasmon holography with white-light illumination. *Science* **332**, 218–220 (2011).

45. J. T. Sheridan, R. K. Kostuk, A. F. Gil, Y. Wang, W. Lu, H. Zhong, Y. Tomita, C. Neipp, J. Francés, S. Gallego, I. Pascual, V. Marinova, S. H. Lin, K. Y. Hsu, F. Bruder, S. Hansen, C. Manecke, R. Meisenheimer, C. Rewitz, T. Rölle, S. Odinokov, O. Matoba, M. Kumar, X. Quan, Y. Awatsuji, P. W. Wachulak, A. V. Gorelaya, A. A. Sevryugin, E. V. Shalymov, V. Yu Venediktov, R. Chmelik, M. A. Ferrara, G. Coppola, A. Márquez, A. Beléndez, W. Yang, R. Yuste, A. Bianco, A. Zanutta, C. Falldorf, J. J. Healy, X. Fan, B. M. Hennelly, I. Zhurminsky, M. Schnieper, R. Ferrini, S. Fricke, G. Situ, H. Wang, A. S. Abdurashitov, V. V. Tuchin, N. V. Petrov, T. Nomura, D. R. Morim, K. Saravanamuttu, Roadmap on holography. *J. Opt.* **22**, 123002 (2020).

46. G. Kawamura, Ag-doped inorganic–organic hybrid films for rewritable hologram memory application. *J. Sol-Gel Sci. Technol.* **79**, 374–380 (2016).

47. C. Li, L. Cao, Z. Wang, G. Jin, Hybrid polarization-angle multiplexing for volume holography in gold nanoparticle-doped photopolymer. *Opt. Lett.* **39**, 6891–6894 (2014).

48. G. Osnabrugge, R. Horstmeyer, I. N. Papadopoulos, B. Judkewitz, I. M. Vellekoop, Generalized optical memory effect. *Optica* **4**, 886–892 (2017).

49. J. Yang, Y. Shen, Y. Liu, A. S. Hemphill, L. V. Wang, Focusing light through scattering media by polarization modulation based generalized digital optical phase conjugation. *Appl. Phys. Lett.* **111**, 201108 (2017).

50. Y. Liu, C. Ma, Y. Shen, J. Shi, L. V. Wang, Focusing light inside dynamic scattering media with millisecond digital optical phase conjugation. *Optica* **4**, 280–288 (2017).

51. X. B. Hu, M. X. Dong, Z. H. Zhu, W. Gao, C. Rosales-Guzmán, Does the structure of light influence the speckle size? *Sci. Rep.* **10**, 199 (2020).

52. Bayfol® HX200 description and application information (2018), (available at https://solutions.covestro.com/)
